# A new allergic rhinitis therapy (MP29-02*) provides nasal and ocular symptom relief days faster than current firstline monotherapies

**DOI:** 10.1186/2045-7022-5-S4-P34

**Published:** 2015-06-26

**Authors:** Peter Hellings, Claus Bachert, Ralph Mösges, Glenis Scadding, Ullrich Munzel, Wytske Fokkens

**Affiliations:** 1 University Hospitals Leuven, Dept of Otorhinolaryngology, Head & Neck Surgery, Leuven, Belgium; 2 Ghent University Hospital, Department of Oto-Rhinolaryngology, Ghent, Belgium; 3 University of Cologne, IMSIE, Cologne, Germany; 4 The Royal National Throat Nose and Ear Hospital, London, UK; 5 Meda, Corporate Clinical Affairs, Bad Homburg, Germany; 6 Academic Medical Center, Department of Otorhinolaryngology, Amsterdam, Netherlands

## Background

The efficacy of MP29-02* (a novel intranasal formulation of azelastine hydrochloride [AZE] and fluticasone propionate [FP] in an advanced delivery system) in providing overall nasal and ocular symptom relief vs AZE, FP or placebo (PLA) has been assessed.

## Methods

Six hundred and ten moderate-to-severe SAR patients (≥12 yrs) were randomized into a double-blind, PLA-controlled, 14-day, parallel-group, trial (NCT00660517) to MP29-02*, AZE, FP or PLA nasal sprays (1 spray/nostril bd; daily dose: AZE=548μg; FP=200μg) [1]. Change from baseline (CFB) in reflective total of 7 symptom scores (rT7SS; AM + PM, max=42) was assessed post-hoc. CFB in rT7SS and each nasal (congestion, itching, rhinorrhea, sneezing) and ocular symptom (itching, redness, watering; max=6 each) was assessed over time.

## Results

Overall, MP29-02* patients showed greater reduction in rT7SS vs FP, AZE & PLA (relative diff: 52% to FP (p=0.0013), 56% to AZE (p=0.0004)) evident from treatment day 1 vs FP (p=0.0072), AZE (p=0.0336) or PLA (p<0.0001) and sustained for 14 days. The level of relief achieved by MP29-02* patients on Day 2 (-5.52) was not achieved before Day 5 by FP patients or Day 8 by AZE patients. This pattern of rapid, sustained and superior symptom relief by MP29-02* was observed for each nasal and ocular symptom, which was not the case with FP and AZE. MP29-02* provided significantly superior nasal congestion relief than FP or AZE from Day 2; the level of congestion relief provided by MP29-02* on Day 2 (-0.77) was not achieved before Day 6 and Day 9 for FP and AZE, respectively. MP29-02* provided significantly superior ocular itching relief vs FP from Day 2 and vs AZE from Day 3; the ocular itch relief provided by MP29-02* on Day 2 (-0.77) was not achieved before Day 10 for FP-patients. Similarly, the level of ocular itch relief provided by MP29-02* on Day 3 (-1.06) was not achieved by AZE-patients before Day 11.

## Conclusion

The consistent and rapid effect in alleviating all nasal and ocular symptoms is unique to MP29-02* and contributes to its superiority over AZE and FP. The time advantage over firstline therapy in achieving significant relief and sustained effect should improve patient concordance. MP29-02* is considered a new standard of care in AR.

* Dymista
